# Direct estimation of the global distribution of vertical velocity within cirrus clouds

**DOI:** 10.1038/s41598-017-07038-6

**Published:** 2017-07-28

**Authors:** Donifan Barahona, Andrea Molod, Heike Kalesse

**Affiliations:** 1 Global Modeling and Assimilation Office, NASA Goddard Space Flight Center, Greenbelt, MD USA; 2 Leibniz Institute for Tropospheric Research, Leipzig, Germany

## Abstract

Cirrus clouds determine the radiative balance of the upper troposphere and the transport of water vapor across the tropopause. The representation of vertical wind velocity, *W*, in atmospheric models constitutes the largest source of uncertainty in the calculation of the cirrus formation rate. Using global atmospheric simulations with a spatial resolution of 7 km we obtain for the first time a direct estimate of the distribution of *W* at the scale relevant for cirrus formation, validated against long-term observations at two different ground sites. The standard deviation in *W*, *σ*_*w*_, varies widely over the globe with the highest values resulting from orographic uplift and convection, and the lowest occurring in the Arctic. Globally about 90% of the simulated *σ*_*w*_ values are below 0.1 m s^−1^ and about one in 10^4^ cloud formation events occur in environments with *σ*_*w*_ > 0.8 m s^−1^. Combining our estimate with reanalysis products and an advanced cloud formation scheme results in lower homogeneous ice nucleation frequency than previously reported, and a decreasing average ice crystal concentration with decreasing temperature. These features are in agreement with observations and suggest that the correct parameterization of *σ*_*w*_ is critical to simulate realistic cirrus properties.

## Introduction

Cirrus clouds, made of ice crystals and present at low temperatures (below 235 K) and high altitudes, cover approximately 17% of the Earth^1^. They control the hydrological balance of the upper troposphere and the longwave cloud feedback^2, 3^. Outside of areas of strong convection, cirrus form by ice nucleation on aerosol particles. Human activities (e.g., transportation and energy production) and natural phenomena (e.g., volcanic eruptions and dust storms) that modify the concentration of atmospheric aerosol influence the properties of cirrus^4^, impacting the hydrological balance of the atmosphere and ultimately Earth’s climate^2, 5–7^. For example, observational studies suggest that volcanic ash leads to the increase of ice-containing clouds in the upper troposphere^6, 7^. There is also evidence of aerosol particles affecting the dehydration efficiency of cirrus in the tropical tropopause, hence the concentration of water vapor entering the stratosphere^8^. Cirrus modification has been suggested as a geoengineering strategy to counterbalance greenhouse warming^9^. However, modeling studies show discrepancy on the predicted response of cloud properties to the emission of distinct aerosol species^5, 10, 11^, introducing uncertainty in the reliability of cirrus geoengineering on climate^12^.

The representation of cirrus in general circulation models (GCMs) remains challenging. Wind velocity, temperature, relative humidity and the physicochemical properties of the aerosol may vary at smaller scales (~1 to 20 km) than the typical resolution used in GCMs (~100–200 km)^13, 14^. Moreover, the ice-nucleating efficiency of aerosol depends on particle size and chemistry, and is not completely understood^15^. Ice formation is also realized by two different processes: homogeneous ice nucleation (HOM), i.e., the spontaneous freezing of supercooled liquid droplets (typically sulfuric acid and sulfate solutions), and, heterogeneous ice nucleation (HET), which requires the presence of ice nucleating particles (INP). Although sparse, INP can profoundly alter the evolution of clouds^5^. Much investigation has been devoted over the last decade to elucidating the nature of INP showing that a small fraction of atmospheric aerosol (typically some organics, mineral dust and black carbon) are capable of nucleating ice^15^. The HOM and HET mechanisms can occur simultaneously, and in fact they “compete” during cloud formation. HOM nucleation requires higher relative humidity (RH) than HET. Thus, by depleting the increase in RH from cooling driven by vertical motion, ice crystals formed by the HET mechanism may inhibit and even prevent HOM nucleation^16^. However, compared to HOM, HET nucleation leads to lower ice crystal concentration^17, 18^.

Vertical motion determines the maximum relative humidity in a cloudy parcel and drives ice nucleation^19^. Early studies showed that introducing vertical velocity perturbations within parcel model simulations resulted in high variability in ice crystal concentration, *N*_*i*_^20, 21^, which has been confirmed by aircraft observations within cirrus^22–24^. Modeling studies have also shown that low *T* and high *W* favors the HOM over the HET mechanism^25–29^, which is reflected in the global distribution of *N*_i_ simulated in GCMs^5, 11, 30^. Field measurements however suggest predominance of HET and lower *N*_i_ than implied by HOM^10, 14, 24^, consistent with sustained levels of supersaturation found in cirrus clouds^23, 31, 32^. This suggests that the frequency of HOM in cirrus is likely overestimated by GCMs. Recent studies point at overestimation in the parameterized cloud-scale *W* as the likely cause of the discrepancy between models and observations^33–36^.

Because of the separation between the relevant scale for ice nucleation and the scale resolved by the GCMs, it is likely that several cloud formation events occur within a model grid cell. This unresolved variability is characterized by a “subgrid” distribution of vertical velocity, ${\rm{\Phi }}(\bar{W},{\sigma }_{w})$, largely determined by its standard deviation, *σ*_*w*_, since the mean resolved vertical transport is slow compared to cloud-scale motions^13, 14, 29^. The latter are typically not resolved by CGMs due to their coarse resolution (~100 km). To represent subgrid *W* variability, most GCMs rely on either poorly constrained parameterizations or empirical correlations representing particular cloud realizations. A recent study^37^ suggested that vertical wind velocity may be responsible for about 90% variation in calculated ice crystal formation rates, although it is not clear whether the same relation applies to other cloud properties. Field campaign analyses and cloud modeling studies^24, 29, 38^ also suggest a strong relation between the effect of aerosol emissions on cloud properties and *W*. These highlight the importance of improving the representation of subgrid *W* variability in GCMs.

In this work we develop a method, using ultra high resolution global simulations to directly calculate the global distribution of subgrid vertical velocity affecting cirrus formation. By implementing our estimates in a global model and running experiments constrained by observations we show that the global distribution of *σ*_*w*_ largely determines the balance between homogeneous and heterogeneous ice nucleation during the formation of cirrus.

## Methods

### Vertical velocity distribution

We developed a method to calculate ${\rm{\Phi }}(\bar{W},{\sigma }_{w})$ using results from a global climate simulation carried out using the non-hydrostatic version of the NASA Goddard Earth Observing System (GEOS-5) over the period 2005–2007^39–41^. The simulation was completed using a cubed-sphere grid with a nominal spatial resolution of 7 km and 72 vertical levels, extending from the surface to 0.1 mbar. The time step for integration was set to five minutes and three-dimensional instantaneous meteorological fields were saved every hour. The vertical resolution of the model in the upper troposphere is about 0.5 km. This simulation is referred as the GEOS-5 “nature run” (G5NR). The output of the simulation amounts to about four petabytes and is used to perform Observation System Simulation Experiments^42^. The G5NR is capable of resolving mesoscale systems and organized convection within large scale midlatitude cyclones, important for the simulation of cirrus^40^.

The global distribution of vertical velocity was calculated collocating *W* from the 7 km G5NR output to 1° (~100 km) horizontal resolution (except for the validation studies, for which 0.5° sections were used) so that at least 256 *W* values from the G5NR were used to calculate *σ*_*w*_ for each 1° grid cell. This procedure resulted in a hourly, three-dimensional global characterization of ${\rm{\Phi }}(\bar{W},{\sigma }_{w})$ at the 1° (~100 km) resolution for the two-year period of the simulation. Monthly averages were calculated by averaging ${\sigma }_{w}^{2}$ using hourly output. Sensitivity tests were carried calculating *σ*_*w*_ for 200 km global resolution, producing essentially similar results as our 100 km calculation, indicating that very little of the total *W* variance is resolved at scales greater than 100 km. A second sensitivity test was performed collocating results from a short term 3.5 km resolution run spanning 15 days in May 2006 to the 1° spatial resolution. After scaling was applied (*α*_1,0_ = 1.26, as explained below, Eq. 4), this test produced similar *σ*_*w*_ as when the 7 km simulation was used (Supplemental Fig. S1).

The G5NR resolves waves and vertical motion with periods from minutes to a few hours which are the main drivers of cirrus formation^22^. However, high frequency waves with periods of a few minutes are responsible for the formation of “pockets” of high *N*_*i*_ within clouds^14, 23^. The scale of such waves would likely be smaller than the 7 km resolution of the G5NR. To account for such a unresolved variability a “scaling” method is developed, as follows. The total spatial vertical velocity variance at the 100 km resolution, ${\sigma }_{w}^{2}$, is represented as the sum of the resolved and unresolved components of the high resolution run,

1 $${\sigma }_{w}^{2}={\sigma }_{w\mathrm{,7}{\rm{km}}}^{2}+{\sigma }_{w,{\rm{unres}}}^{2},$$

where ${\sigma }_{w\mathrm{,7}{\rm{km}}}^{2}$ is the spatial variance in *W* calculated from the 7 km resolution output, and ${\sigma }_{w,{\rm{unres}}}^{2}$ the variance resulting from vertical motion with characteristic scales below 7 km. The contribution to the *W* spatial variability from motion with scales greater than 100 km is neglected. This is justified because motion with scales greater than 100 km is resolved at the low resolution. To estimate ${\sigma }_{w,{\rm{unres}}}^{2}$, the approach of Pauluis, *et al*.^43^ is employed. Using a discretized version of the equations of motion of an anelastic fluid, the authors derived a relation for the vertical velocity resolved at two different horizontal resolutions, e.g., *r*_0_ and *r*_1_,

2 $$\frac{{W}_{{r}_{0}}}{{W}_{{r}_{1}}}={\alpha }_{\mathrm{1,0}}={(\frac{1+\frac{{r}_{1}}{{\rm{\Delta }}Z}}{1+\frac{{r}_{0}}{{\rm{\Delta }}Z}})}^{\mathrm{1/2}},$$

where Δ*Z* = 6 km corresponds to the resolution at which the increase in kinetic energy from buoyancy is equally distributed between its horizontal and vertical components. Pauluis, *et al*.^43^ showed that Eq. (2) is accurate for horizontal resolutions between 2 and 20 km, hence it is suitable to scale the G5NR, 7 km results, to finer resolutions, hence to estimate the variance unresolved by the G5NR. In this study we assume *r*_0_ = 0.1 km as the horizontal scale below which the cloud properties can be considered uniform. Using *r*_1_ = 7 km (i.e., the resolution of the G5NR) results in *α*_1,0_ = 1.46. For *r*_1_ = 3.5 km, *α*_1,0_ = 1.26. The choice of *r*_0_ is rather arbitrary, however for small enough values has little effect on *α*_1,0_ (e.g., *α*_1,0_ = 1.41 for *r*_0_ = 0.5 km). Here *r*_0_ is selected much smaller than the typical cloud size to account for short-lived fluctuations that may affect the relaxation of supersaturation in the cloud^14, 29, 44, 45^.

For the special case of a normal distribution at resolution *r*_1_,

3 $${W}_{{r}_{1}}\sim {\rm{\Phi }}\,(\bar{W},{\sigma }_{w\mathrm{,7}{\rm{km}}})$$

where $\bar{W}$ is the grid-scale vertical velocity. Combining Eqs (2) and (3) it can be readily shown that,

4 $${W}_{{r}_{0}}\sim {\rm{\Phi }}\,({\alpha }_{\mathrm{1,0}}\bar{W},{\alpha }_{\mathrm{1,0}}{\sigma }_{w\mathrm{,7}{\rm{km}}}).$$

Which implies *σ*_*w*_ = *α*_1,0_ *σ*_*w*,7km_. Equation (4) provides a simple argument to scale the resolved variance calculated directly from the G5NR output, ${\sigma }_{w\mathrm{,7}{\rm{km}}}^{2}$, to obtain the total variance driving cloud formation, ${\sigma }_{w}^{2}$, at the 100 km resolution.

### Validation

The vertical velocity fields from the 7 km simulation were validated against ground-based measurements within cirrus for two different sites with diverse topography and synoptic conditions, and for which long term radar retrievals are available^13^. These correspond to the Southern Great Plains of North America (SGP, 36° 36′ 18″ N, 97° 29′ 6″ W) and the Manus island in the tropical western Pacific (Manus, 2° 3′ 39.64″ S, 147° 25′ 31.43″ E). SGP is a mid-latitude continental site with large variability in temperature and cloud occurrence. Manus is an oceanic site off the coast of Australia with frequent tropical convection. Ground-based radar retrievals^13^ of vertical velocity at each site over the period (2000–2010) were obtained from the Atmospheric Radiation Measurement program (www.arm.gov/sites). The retrieval algorithm is based on a decomposition of the Doppler vertical velocity. The typical error in vertical velocity is about 15 cm s^−1^ for a minimum reflectivity of about −40 dBz^13^. The whole data set spans about 10 years for each site. Data obtained at 10 s intervals for each month were averaged over 5 min to match the time step of the G5NR simulation.

**Figure 1:**
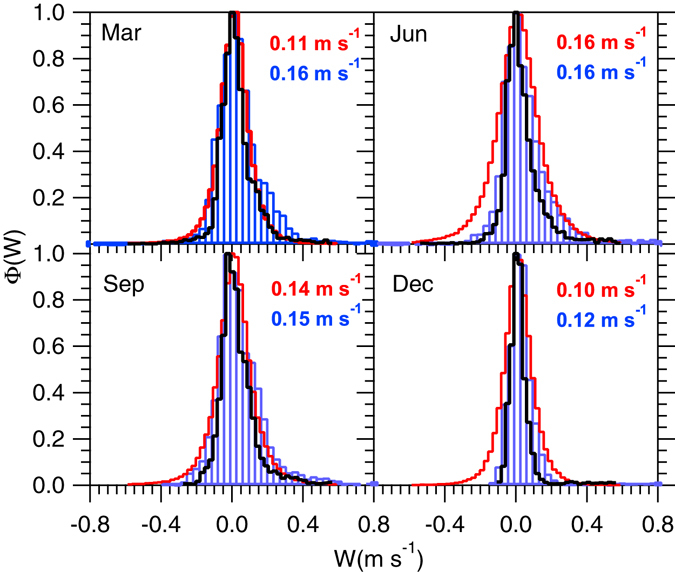
Vertical velocity (m s^−1^) distribution within cirrus at the SGP site from cloud Doppler radar measurements^13^ (red lines) and high resolution simulations (blue bars). Measurements correspond to the period 2000–2010 and model results to 2005–2007. Scaling was applied to the model results as described in Methods. Black lines represent the “unscaled distribution” from the G5NR. Positive *W* values indicate updraft. The legends correspond the mean value of *σ*_*w*_ for each case.

**Figure 2:**
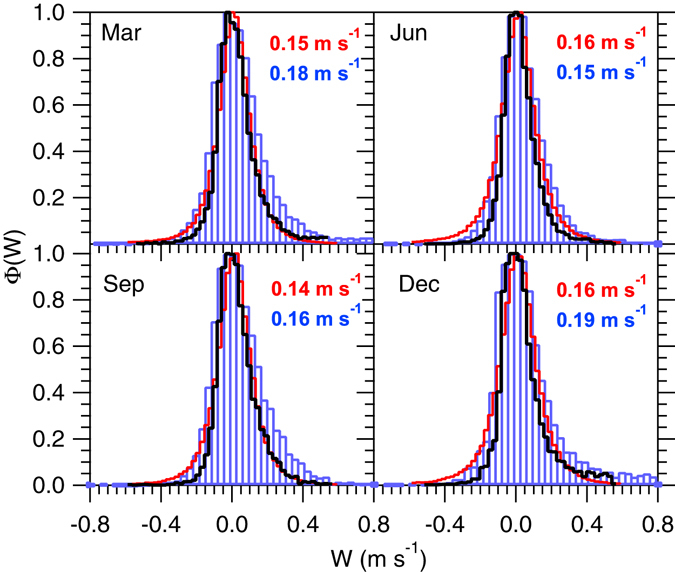
Similar to Fig. 1 but for the Manus site.

To generate G5NR vertical velocity distributions at the SGP (Fig. 1) and Manus (Fig. 2) sites, instantaneous *W* values over a 0.5° × 0.5° area centered at each site were obtained from the 7 km simulation at three hour intervals. This corresponds to about 15360 values for each monthly distribution at each site. Notice that this includes the spatial and temporal components of the variance since it is difficult to separate them in the observations. The model results were restricted to ice mixing ratios above 5 × 10^−5^ kg kg^−1^ and corresponding ice water content of about 50 mg m^−3^, selected to match the maximum sensitivity of the retrieval method^13^.

To account for vertical motion with scales below 7 km, likely resulting from *in situ* turbulence and high-frequency gravity waves^13^, the distributions were scaled to 0.1 km horizontal resolution using the method outlined in the previous section. Equation (2) was applied directly to the simulated distributions, i.e., without invoking the assumption of a normal ${\rm{\Phi }}(\bar{W},{\sigma }_{w})$. However Figs 1 and 2 suggest that ${\rm{\Phi }}(\bar{W},{\sigma }_{w})$ can be adequately approximated using Gaussian functions, in line with literature reports^26, 29, 46–48^.

Comparison of the measured and simulated ${\rm{\Phi }}(\bar{W},{\sigma }_{w})$ (Figs 1 and 2) shows that the G5NR is capable of generating realistic vertical velocity distributions, and in reasonable agreement with observations. At the SGP site it is evident that using only the raw G5NR data would result in a too-narrow ${\rm{\Phi }}(\bar{W},{\sigma }_{w})$ compared to the measurements (Fig. 1, black lines). Scaling brings ${\rm{\Phi }}(\bar{W},{\sigma }_{w})$ closer to the observations suggesting that a significant fraction of the *W* variance lies in the sub-7 km range. On the other hand, for the Manus site the raw G5NR distribution (Fig. 2, black lines) approximates the observed distribution (Fig. 2, red lines), indicating that most of the *W* variance is resolved at the 7 km resolution. In the latter, scaling may lead to overestimation in *σ*_*w*_ since Eq. (2) implicitly assumes that a significant fraction of the *W* variance lies at the small scales. Thus, *σ*_*w*_ can be considered a upper limit to the vertical velocity variance.

**Figure 3:**
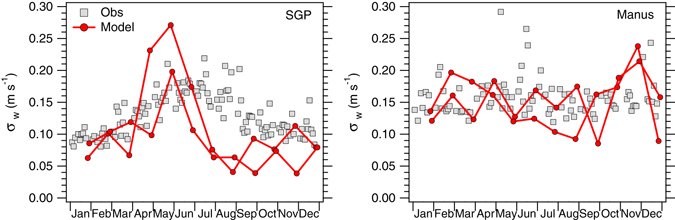
Monthly mean standard deviation in vertical velocity (m s^−1^) at the SGP and Manus sites calculated from 7 km global output (model) and from radar retrievals (obs). Error bars have been omitted for clarity however the standard error in the observations^13^ is about 0.15 m s^−1^.

Seasonal variation in the large scale environment may lead to differences between the simulated and observed distributions. Such deviations are typically within the margin of error of the observations. However they can also signal systematic errors in the simulated ${\rm{\Phi }}(\bar{W},{\sigma }_{w})$. To study the latter, *σ*_*w*_ was calculated for each month over the whole observational record (resulting in ten data points per month) and from the G5NR (which are available for two separate years). The results are plotted in Fig. 3. At both sites, the simulated and observed *σ*_*w*_ show little interannual variability, i.e., for the same month the spread in *σ*_*w*_ between years is typically below 0.05 m s^−1^. *σ*_*w*_ at the SGP site shows a strong annual cycle, whereas at the Manus site it is relatively constant over the year. This suggests that location plays an important role in determining *σ*_*w*_. The distinctive behavior of *σ*_*w*_ at each site is well represented by the simulation. However the G5NR tends to predict a stronger annual cycle of *σ*_*w*_ at the SGP site than implied by observations, with the maximum *σ*_*w*_ occurring too early during the year and an underestimation in *σ*_*w*_ between August and October, likely related to the low frequency of convective events during the Fall season in the G5NR^40^.

### GCM implementation

The calculated *σ*_*w*_ was used to drive ice nucleation in GEOS-5 and study the impact on the balance between HOM and HET processes. Due to the high computational expense of the 7 km simulations, aerosol-cloud interactions are analyzed in the lower resolution, 100 km, simulation, but using *σ*_*w*_ obtained from the 7 km run. The goal of the GCM implementation is to analyze the statistics of the ice crystal concentration when the G5NR-generated distribution of *σ*_*w*_ is used. The main premises behind this approach are that the interannual variability in *σ*_*w*_ is small and that *σ*_*w*_ is mostly influenced by local features and convection. This is supported by the low interannual variability in *σ*_*w*_ found in our 7 km simulation, and in the 10-year-long time series of radar retrievals at the SGP and Manus sites (Fig. 3).

The model’s cloud microphysics and ice nucleation schemes are described elsewhere^49^. Briefly, HOM occurs on sulfate whereas mineral dust, black carbon and glassy organics are considered INP^50^. The ice nucleation rate is weighted by the distribution of vertical velocity and explicitly integrated. Large scale deposition, ice cloud fraction, and ice nucleation are coupled^49^. To minimize the effect of model uncertainties from the transport of aerosol and the meteorological conditions, the 100 km simulation is constrained using the horizontal wind velocity, temperature and water vapor from the second version of the Modern-Era Retrospective Analysis for Research and Applications (MERRA-2)^39, 51^, using a relaxation time scale of 6 h. Monthly-averaged *σ*_*w*_ derived from the G5NR was used to drive ice nucleation. To perform ice nucleation studies, the model was run for five years (2005–2010) using a c90 cubed-sphere grid (spatial resolution of about 1°) and 72 vertical levels. Cloud physical and optical properties obtained in similar simulations have been shown to be in agreement with available *in situ* and satellite observations^49^.

A cirrus formation event was considered dominated by homogeneous freezing when at least 80% of the ice crystals were produced by the HOM mechanism. Modeling studies^17, 18^ show that cloud formation becomes HET dominated within a relative narrow range of INP concentration, typically less than a factor of two. Thus the 80% limit represents the INP concentration at which *N*_*i*_ becomes strongly affected by HET nucleation. Results using different thresholds, i.e., 20%, 50% and 90% are discussed below. Bivariate *N*_*i*_ vs. *T* distributions were calculated by counting the number of data points falling within a particular *N*_*i*_ and *T* combination, using 80 logarithmic bins for *N*_*i*_ and 80 linear bins for *T*. The frequencies were then normalized by the most frequent *N*_*i*_ vs. *T* combination within the entire domain. Using this method the expected value of *N*_*i*_ is located around the maximum frequency at each temperature.

### Data Availability

The GEOS-5 source code is available under the NASA Open Source Agreement http://opensource.gsfc.nasa.gov/projects/GEOS-5/.

## Results

### Vertical velocity distribution

**Figure 4:**
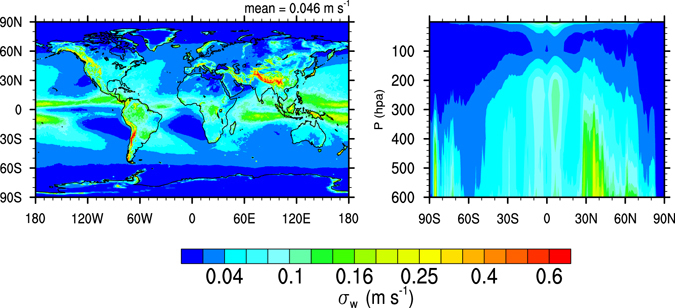
Annual mean standard deviation in vertical velocity (m s^−1^) at a horizontal resolution of 1°, calculated from 7 km global output. Left panel: *σ*_*w*_ at 250 hPa. Right panel: zonal mean *σ*_*w*_. Maps generated using the NCAR Command Language (Version 6.3.0) Software. (2016). Boulder, Colorado: UCAR/NCAR/CISL/TDD. http://dx.doi.org/10.5065/D6WD3XH5.

Figure 4 shows the annual global distribution of *σ*_*w*_ derived from the G5NR. High values of *σ*_*w*_ are found around the Inter-Tropical Convergence Zone (ITCZ) and over continental mountain ranges, confirming the notion that underlying convection and orography are the main drivers of dynamic variability in the upper troposphere^46, 49, 52^. For the same reason *σ*_*w*_ is typically higher in the Subtropical Northern hemisphere (NH) than in the Southern hemisphere (SH), except over the Andes mountains where *σ*_*w*_ tends to be high due to orographic uplift. Within the troposphere *σ*_*w*_ decreases slightly with height, and becomes small above the tropopause due to gravity wave breaking. This is in agreement with observations^53^ however differs from previous work where *W*, instead of *σ*_*w*_, was assumed to decrease with height^54, 55^. Figure 4 suggests that high values of *W* do occur at high altitude, but they become less frequent near the tropopause since ${\rm{\Phi }}(\bar{W},{\sigma }_{w})$ becomes narrow.

Aside from the poles, subtropical regions, usually associated with persistent stratocumulus decks and large scale subsidence (i.e., the coasts of Peru, California and Namibia), display the smallest *σ*_*w*_ over the globe. These areas are also associated with a low frequency of cirrus clouds^1^. Low *σ*_*w*_ results in low *N*_*i*_ and large ice settling velocities, which may contribute to a more efficient cloud removal and explain in part the low cirrus frequency of these regions. At constant pressure *σ*_*w*_ tends to decrease poleward from the Tropics in both hemispheres due in part to the breaking of gravity waves at higher pressure levels. In NH the lowest *σ*_*w*_ values are found at the northernmost latitudes (above 80°). In SH the lowest values of *σ*_*w*_ are found around −60°, however *σ*_*w*_ increases south of −60° revealing the orographic effect of the Antarctic continent on *W*, which may impact the formation of polar stratospheric clouds.

**Figure 5:**
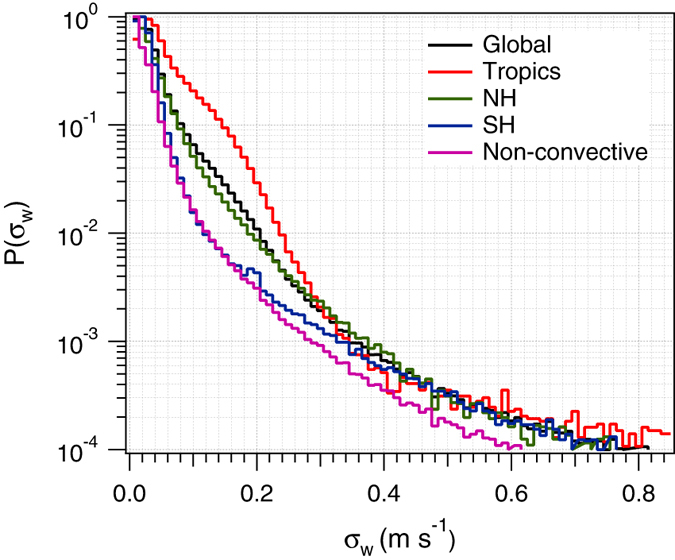
Probability distribution of *σ*_*w*_ for different regions, and for grid cells with no underlying convection.

Figure 5 shows the global PDF of *σ*_*w*_, *P*(*σ*_*w*_), within cirrus, i.e., positive ice water content. As expected, *P*(*σ*_*w*_) decreases exponentially with *σ*_*w*_. However several features of *P*(*σ*_*w*_) stand out. *P*(*σ*_*w*_) peaks at around 0.02 m s^−1^ and decreases rapidly with increasing *σ*_*w*_ so that about 90% of the values of *σ*_*w*_ are below 0.1 m s^−1^. This suggests that in most cases cirrus formation is driven by large scale vertical transport and inertial gravity waves. Higher values of *σ*_*w*_ (up to 0.8 m s^−1^) are associated with high frequency gravity waves from convective and orographic sources^56^. Although likely, they are progressively less frequent as *σ*_*w*_ increases. Globally about one in 10^4^ non-convective cirrus formation events are forced by vertical motion with *σ*_*w*_ > 0.8 m s^−1^. High values of *σ*_*w*_ are more likely in the Tropics driven by underlying convection. Removing grid-cells with vigorous convection results in about a factor of five lower probability of finding *σ*_*w*_ > 0.2 m s^−1^. Values of *σ*_*w*_ greater than 0.5 m s^−1^ seem to be equally likely in SH and NH indicating that they may result from strong orographic uplift in the mountain ranges.

**Figure 6:**
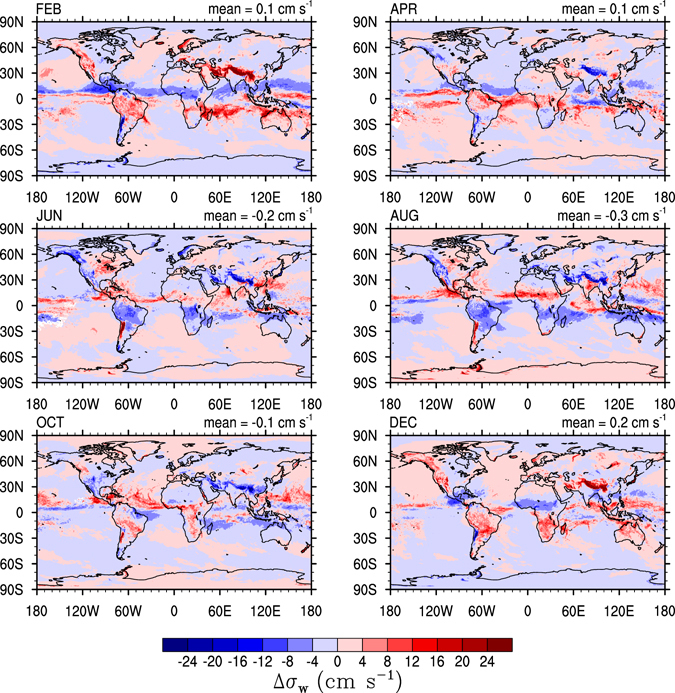
Difference between the monthly and annual *σ*_*w*_ at 100 km resolution calculated from the G5NR 7 km resolution output at the 250 hPa pressure level. Maps generated using the NCAR Command Language (Version 6.3.0) Software. (2016). Boulder, Colorado: UCAR/NCAR/CISL/TDD. http://dx.doi.org/10.5065/D6WD3XH5.

There is a slight seasonal variation in the global distribution of *σ*_*w*_. Over the Tropics the highest *σ*_*w*_ coincides with the displacement of the ITCZ (Fig. 6). Over the continents and away from orographic features, *σ*_*w*_ is influenced by large scale meteorological patterns, i.e., cold fronts, midlatitude cyclones, and subtropical jets^57^. In the subtropics *σ*_*w*_ tends to be larger during the summer months, particularly over land. The two years of the G5NR simulation show similar patterns: highest in the ITCZ and in orographic regions and lowest in the high latitudes with little variation in the Subtropical regions. Location seems to be the main factor determining *σ*_*w*_. This is supported by the data at the Manus and SGP sites (Fig. 3), and it is in line with literature reports showing low interannual variability in ${\rm{\Phi }}(\bar{W},{\sigma }_{w})$ for the same location^48, 57^. A longer simulation period is however required to further study the interannual variability in ${\rm{\Phi }}(\bar{W},{\sigma }_{w})$ and will be the subject of a future study.

The results shown in Fig. 4 can be used to evaluate the parameterization of *σ*_*w*_ currently used in GEOS-5 (Barahona *et al*.^49^, c.f. Fig. 4). Compared to the parameterized results, *σ*_*w*_ is lower in the marine midlatitudes and higher in the Tropics. This parameterization used the orographic stress and turbulence to estimate *σ*_*w*_. Over the ocean the total surface stress was used, leading to an overestimation of *σ*_*w*_ in the midlatitudes. On the other hand, the parameterization did not explicitly account for gravity waves generated by convection leading to underestimation of *σ*_*w*_ in the Tropical region. Over land the agreement is better since orographic features drive gravity wave generation^52^. As similar parameterizations are used in many GCMs, this exercise shows that Fig. 4 may provide a way to evaluate their performance.

### Implications for ice cloud formation

**Figure 7:**
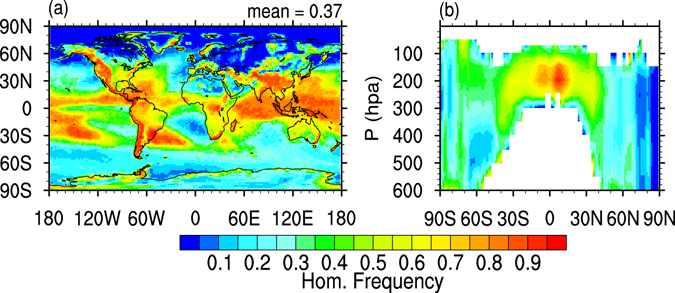
Global distribution of the frequency of cirrus events dominated by homogeneous ice nucleation, vertically weighted by cloud fraction (**a**) and zonal mean (**b**). Maps generated using the NCAR Command Language (Version 6.3.0) Software. (2016). Boulder, Colorado: UCAR/NCAR/CISL/TDD. http://dx.doi.org/10.5065/D6WD3XH5.

The annual mean frequency of homogeneous freezing events during cirrus formation calculated with GEOS-5 (forcing ice nucleation with *σ*_*w*_ calculated from the G5NR output) is shown in Fig. 7. High *σ*_*w*_ and low *T* tend to lead to high annual HOM frequency. This is because under such conditions a larger INP concentration is required to offset the increase in RH from expansion cooling, and is evidenced by the correspondence in the spatial patterns seen in Figs 4 and 7. In the Tropical region where high values of *σ*_*w*_ are more likely, HOM dominates about 50–60% of the cloud formation events, and up to 80% in the coldest regions of the Tropical tropopause, over the Central Pacific, where a lack of INP also contributes to diminish the frequency of HET nucleation. Outside the Tropical and Subtropical regions, the frequency of cloud events dominated by HOM nucleation is generally low (below 30%), particularly in the NH. In fact, in the Arctic clouds tend to form almost exclusively by HET (HOM frequency is below 10%), mostly resulting from the low *σ*_*w*_ which precludes efficient HOM nucleation, even though the INP availability is low. HOM-dominated cirrus events are also less frequent over North Africa due to the presence of dust and black carbon, and to the absence of orographic features and convection that would produce high *σ*_*w*_.

**Figure 8:**
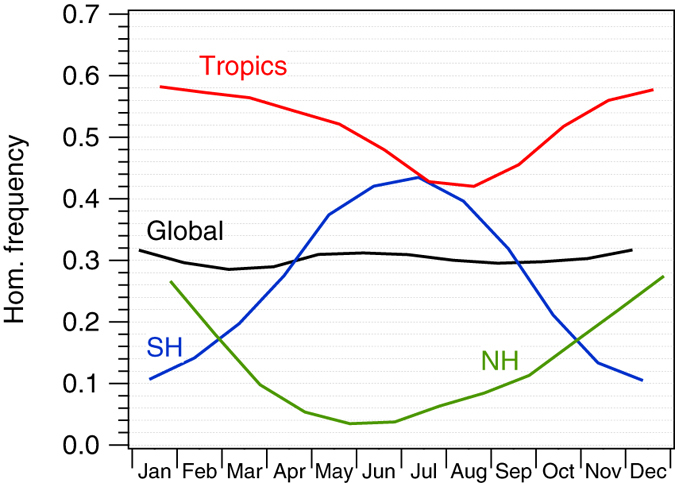
Monthly mean homogeneous ice nucleation frequency for the Tropical (latitude −30° to 30°) and the Northern (NH, latitude 30° to 60°), and Southern (SH, latitude −30° to −60°) extratropical regions.

While the global mean HOM-dominated frequency is relatively constant over the year at ~37%, in both the SH and NH it peaks in the winter months, since low temperature favors HOM (Fig. 8). Maximum HOM occurrence in the SH is 45% during the winter months, while it is only 30% in the NH winter. Even though *σ*_*w*_ is higher on average in the NH than in the SH, HOM is more prevalent in the latter during most of the year since INP like mineral dust are abundant in the NH^58^. The seasonal differences in the SH are more pronounced than in the NH due to the larger temperature and aerosol variability, and lower *σ*_*w*_ in the former. In the Tropics, a seasonal cycle is also present reflecting the strength and position of the ITCZ influencing *σ*_*w*_ and the annual variation in black carbon and dust concentration.

The balance between HOM and HET during cloud formation is significantly influenced by the concentration of ice nucleating particles, *N*_INP_. Supplementary Figure S2 shows the frequency of *N*_INP_ calculated at the maximum RH during cloud formation, *RH*_max_. *N*_INP_ increases steeply around *RH*_max_ = 15% to a maximum global average value of about 3 L^−1^ at *RH*_max_ = 30% (~5 L^−1^ for NH). The apparent decrease in *N*_INP_ at high *RH*_max_ is caused by competition between HOM and HET nucleation. For *RH*_*max*_ < 40% where HOM does not occur, the simulated *N*_*INP*_ shows similar characteristics as available reports^58^. This is in part by design as the heterogeneous ice nucleation spectrum used in the model is derived from a collection of available field data^50^. Figure S2 shows that GEOS-5 simulates realistic *N*_INP_ statistics.

**Figure 9:**
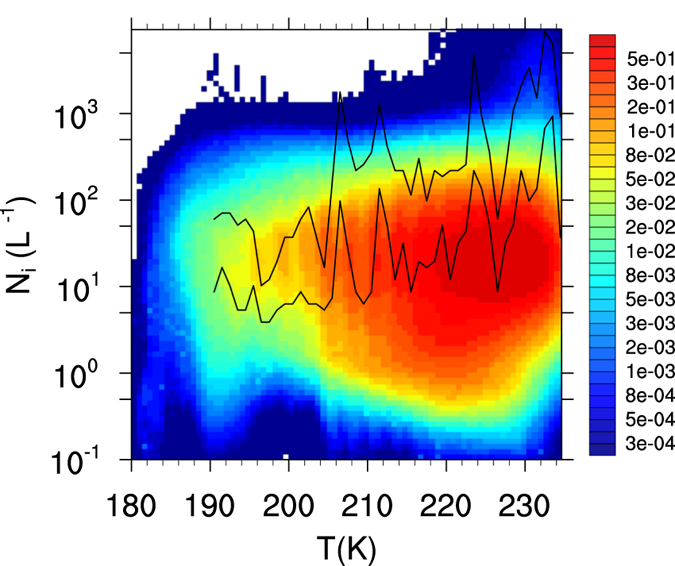
Global frequency distribution of in-cloud ice crystal number concentration as a function of temperature from GEOS-5 output over a 2-year subset (2005–2006). Solid lines represent the 25% and 75% quantiles from a compilation of field campaign observations^23^.

Globally, about 70% of the time cirrus form in situations where HOM and HET occur simultaneously, with HET being more prevalent. This finding is at odds with previous modeling studies where HOM is the predominant cirrus formation mechanism^5, 49, 54^. Our results are however in better agreement with field campaign data suggesting a significant role of dust and other INP species in cirrus formation^10^, and, lower *N*_*i*_ and higher saturation ratios than implied by HOM^8, 14^. This suggests that the parameterization of *σ*_*w*_ may be responsible for the higher HOM frequency typically found in modeling studies. Notably, forcing ice nucleation with our estimate of *σ*_*w*_ also results in good agreement of *N*_*i*_ with field campaign data at very low temperature (Fig. 9, *T* < 200 K), where most modeling studies show high overestimation of *N*_*i*_^5, 36^. This suggests that the overestimation in many models may be a result of poorly constrained *σ*_*w*_. GEOS-5 however seems to overestimate the frequency of low *N*_*i*_ for *T* > 230 K. It must be noticed that ice shattering on *in*-*situ* probes, leading to overestimation in the *in*-*situ N*_*i*_, are a likely artifact of the measurements at such temperatures^59^. Results for the NH, where most cirrus field campaigns have taken place^23^ show similar tendencies (see Supplementary Fig. S3) with slightly lower variability in *N*_*i*_ and better agreement with observations for *T* > 230*K*.

The effect of selecting different thresholds to define a HOM-dominated cirrus is shown in the Supplementary Fig. S4. Changing the definition of a HOM-dominated cloud from 90% to 50% of *N*_*i*_ produced by HOM, increases the HOM frequency from 33% to 43%. The latter corresponds to the minimum threshold where a cloud could be referred as HOM-dominated since for any value below 50% most of the ice crystals would in fact be produced by HET nucleation. Even at the 50% threshold, the HOM frequency is still much lower than reported values showing that our conclusions are not significantly influenced by the selected threshold. Using a more strict definition, where HOM still occurs but is not dominant, i.e., 20% of ice crystals produced by HOM (actually a HET-dominated cloud) leads to global HOM frequency of about 48% which is still low compared to current reports^5, 49^.

## Discussion and Conclusions

This work reports for the first time the direct estimation of the subgrid spatial variance in vertical velocity at the scale relevant for cirrus formation. Figure 4 shows that *σ*_*w*_ has considerable spatial and seasonal variability. It is important for GCM parameterizations of *σ*_*w*_ to reproduce such a spatial variability. This indicates that parameterizations based on individual field campaigns should be used with caution when applied to the global scale. Large-scale dynamics, turbulence, orographic uplift, and underlying convection impact *σ*_*w*_. Even State-of-the-Art parameterizations of *σ*_*w*_^49, 52^ neglect the effect of convection generating gravity waves that can impact cirrus formation, which may result in a too-low frequency of high *σ*_*w*_ as indicated by Fig. 5.

Using the direct estimate of *σ*_*w*_ to drive the GEOS-5 ice nucleation scheme results in a lower predominance of homogeneous ice nucleation than previously simulated, in better agreement with field measurements. Such lower frequency of HOM events also results in a better agreement of simulated *N*_*i*_ with observations, particularly at low *T* (Fig. 9). This is significant as cloud formation theory typically predicts high *N*_*i*_ for low temperature^60, 61^, a trend also found in GCM studies. One way to reconcile theory and observations is to assume very low *W* at low *T* ^30, 36, 54^, which however conflicts with observations of high *W* (>0.5 m s^−1^) near the tropopause^14, 23, 62^. Our results provide a way to reconcile these two views. High values of *W* do occur at low *T*, however the low *σ*_*w*_ at high altitude limits the frequency of cloud formation events driven by high *W*, and on average leads to lower *N*_*i*_. This is agreement with previous work suggesting the structuring of cirrus by dynamics^29, 33, 45^ and an episodic nature of HOM in cirrus^14^.

Our new estimate provides a way to validate new parameterizations of *σ*_*w*_ at a global scale. Several uncertainties however remain in the modeling of cirrus clouds, the most significant being the concentration of INP in the upper troposphere. Even though our results are in relative good agreement with available reports (Supplementary Fig. S2), still few studies provide observations of *N*_*INP*_ with a wide range of aerosol concentrations, temperature and relative humidity and enough spatial coverage that would allow comprehensive validation of GCM predictions. The characterization of *W* at low *T* also requires better techniques with smaller errors, since in some cases they can be as high as *σ*_*w*_^13^. In turn an improved representation of *σ*_*w*_ in GCMs may help reducing the uncertainty surrounding the estimation of the impact of aerosol emissions on cirrus, and eventually lead to a better prediction of future climate.

## Electronic supplementary material

Supplementary Material

## References

[1] Sassen, K., Wang, Z. & Liu, D. Global distribution of cirrus clouds from cloudsat/cloud-aerosol lidar and infrared pathfinder satellite observations (calipso) measurements. *J*. *Geophys*. *Res*.: *Atmospheres* **113**, doi:10.1029/2008JD009972 (2008).

[2] Myhre, G. *et al*. *Anthropogenic and Natural Radiative Forcing*. *Climate Change 2013*: *The Physical Science Basis*. *Contribution of Working Group I to the Fifth Assessment Report of the Intergovernmental Panel on Climate Change*, book section 8, 659–740, www.climatechange2013.org (Cambridge University Press, Cambridge, United Kingdom and New York, NY, USA, 2013).

[3] Zelinka MD, Hartmann DL (2012). Climate feedbacks and their implications for poleward energy flux changes in a warming climate. J. Climate.

[4] Boucher, O. *et al*. *Clouds and Aerosols*. *Climate Change 2013*: *The Physical Science Basis*. *Contribution of Working Group I to the Fifth Assessment Report of the Intergovernmental Panel on Climate Change*, book section 7, 571–658, www.climatechange2013.org (Cambridge University Press, Cambridge, United Kingdom and New York, NY, USA, 2013).

[5] Gettelman, A., Liu, X., Barahona, D., Lohmann, U. & Chen, C. Climate impacts of ice nucleation. *J*. *Geophys*. *Res*. **117**, doi:10.1029/2012JD017950 (2012).

[6] Seifert, P. *et al*. Ice formation in ash-influenced clouds after the eruption of the eyjafjallajökull volcano in april 2010. *J*. *Geophys*. *Res*.: *Atm*. **116**, doi:10.1029/2011JD015702 (2011).

[7] Friberg J (2015). Influence of volcanic eruptions on midlatitude upper tropospheric aerosol and consequences for cirrus clouds. Earth Space Sci..

[8] Schoeberl MR, Selkirk HB, Vömel H, Douglass AR (2015). Sources of seasonal variability in tropical upper troposphere and lower stratosphere water vapor and ozone: Inferences from the ticosonde data set at costa rica. J. Geophys. Res..

[9] Storelvmo T (2013). Cirrus cloud seeding has potential to cool climate. Geophys. Res. Lett..

[10] Cziczo DJ (2013). Clarifying the dominant sources and mechanisms of cirrus cloud formation. Science.

[11] Hendricks J, Kärcher B, Lohmann U (2011). Effects of ice nuclei on cirrus clouds in a global climate model. J. Geophys. Res..

[12] Cirisan A (2013). Microphysical and radiative changes in cirrus clouds by geoengineering the stratosphere. J. Geophys. Res.: Atmospheres.

[13] Kalesse, H. & Kollias, P. Climatology of high cloud dynamics using profiling arm doppler radar observations. *J*. *Climate* **26**, 6340–6359, doi:10.1175/JCLI-D-12-00695.1 (2013).

[14] Jensen EJ (2013). Ice nucleation and dehydration in the tropical tropopause layer. Proc. Nat. Acad. Sci..

[15] Hoose C, Möhler O (2012). Heterogeneous ice nucleation on atmospheric aerosols: a review of results from laboratory experiments. Atm. Chem. Phys..

[16] DeMott P, Meyers M, Cotton R (1994). Parameterization and impact of ice initiation processes relevant to numerical model simulations of cirrus clouds. J. Atmos. Sci..

[17] Kärcher B, Hendricks J, Lohmann U (2006). Physically based parameterization of cirrus cloud formation for use in global atmospheric models. J. Geophys. Res..

[18] Barahona D, Nenes A (2009). Parameterizing the competition between homogeneous and heterogeneous freezing in cirrus cloud formation. monodisperse ice nuclei. Atmos. Chem. Phys..

[19] Pruppacher, H. & Klett, J. *Microphysics of clouds and precipitation*, 2nd edn (Kluwer Academic Publishers, Boston, MA, 1997).

[20] Jensen E, Toon O, Westphal D, Kinne S, Heymsfield A (1994). Microphysical modeling of cirrus 1. comparison with 1986 fire ifo measurements. J. Geophys. Res..

[21] Lin H, Noone K, Ström J, Heymsfield A (1998). Dynamical influences on cirrus cloud formation process. J. Atmos. Sci..

[22] Kärcher, B. & Ström, J. The roles of dynamical variability and aerosols in cirrus cloud formation. *Atm*. *Chem*. *Phys*. **3**, 823–838, http://www.atmos-chem-phys.net/3/823/2003/, doi:10.5194/acp-3-823-2003 (2003).

[23] Krämer M (2009). Ice supersaturation and cirrus cloud crystal numbers. Atmos. Chem. Phys..

[24] Krämer, M. *et al*. A microphysics guide to cirrus clouds–part 1: Cirrus types. *Atm*. *Chem*.*Phys*. **16**, 3463–3483, http://www.atmos-chem-phys.net/16/3463/2016/, doi:10.5194/acp-16-3463-2016 (2016).

[25] Haag W, Kärcher B (2004). The impact of aerosols and gravity waves on cirrus at midlatitudes. J. Geophys. Res..

[26] Jensen E, Pfister L (2004). Transport and freeze-drying in the tropical tropopause layer. J. Geophys. Res..

[27] Spichtinger P, Gierens K (2009). Modeling of cirrus clouds - part 1b: Structuring cirrus clouds by dynamics. Atmos. Chem. Phys..

[28] Jensen E, Pfister L, Bui T-P, Lawson P, Baumgardner D (2010). Ice nucleation and cloud microphysical properties in tropical tropopause layer cirrus. Atm. Chem. Phys..

[29] Barahona D, Nenes A (2011). Dynamical states of low temperature cirrus. Atmos. Chem. Phys..

[30] Barahona D, Rodriguez J, Nenes A (2010). Sensitivity of the global distribution of cirrus ice crystal concentration to heterogeneous freezing. J. Geophys. Res..

[31] Selkirk, H. B. *et al*. Detailed structure of the tropical upper troposphere and lower stratosphere as revealed by balloon sonde observations of water vapor, ozone, temperature, and winds during the nasa tcsp and tc4 campaigns. *J. Geophysi. Res.* **115**, D00J19, http://dx.doi.org/10.1029/2009JD013209, doi:10.1029/2009JD013209 (2010).

[32] Rollins A (2016). Observational constraints on the efficiency of dehydration mechanisms in the tropical tropopause layer. Geophys. Res. Lett..

[33] Spichtinger P, Krämer M (2013). Tropical tropopause ice clouds: a dynamic approach to the mystery of low crystal numbers. Atm. Chem. Phys..

[34] Sheyko, B. *et al*. Quantifying sensitivities of ice crystal number and sources of ice crystal number variability in cam 5.1 using the adjoint of a physically based cirrus formation parameterization. *J*. *Geophys*. *Res*.: *Atm*. **120**, 2834–2854, doi:10.1002/2014JD022457 (2015).

[35] Shi, X. & Liu, X. Effect of cloud-scale vertical velocity on the contribution of homogeneous nucleation to cirrus formation and radiative forcing. *Geophys*. *Res*. *Lett*., doi:10.1002/2016GL069531 (2016).

[36] Zhou, C., Penner, J. E., Lin, G., Liu, X. & Wang, M. What controls the low ice number concentration in the upper troposphere? *Atm*. *Chem*. *Phys*. **16**, 12411–12424, http://www.atmos-chem-phys.net/16/12411/2016/, doi:10.5194/acp-16-12411-2016 (2016).

[37] Sullivan, S. C., Lee, D., Oreopoulos, L. & Nenes, A. Role of updraft velocity in temporal variability of global cloud hydrometeor number. *Proc*. *Natl*. *Acad*. *Sci*. 5791–5796 (2016). 10.1073/pnas.1514039113 PMC4889347 27185952

[38] Kärcher B, Ström J (2003). The roles of dynamical variabilty and aerosols in cirrus cloud formation. Atmos. Chem. Phys..

[39] Molod A, Takacs L, Suarez M, Bacmeister J (2015). Development of the geos-5 atmospheric general circulation model: evolution from merra to merra2. Geosc. Model Dev..

[40] Gelaro, R. *et al*. *Evaluation of the 7*-*km GEOS*-*5 Nature Run*, vol. 36 of *Technical Report Series on Global Modeling and Data Assimilation* (NASA Goddard Space Flight Center, Greenbelt, MD, USA, 2015).

[41] Putman W, Suarez M (2011). Cloud-system resolving simulations with the nasa goddard earth observing system global atmospheric model (geos-5). Geophys. Res. Lett..

[42] Privé, N. & Errico, R. Temporal and spatial interpolation errors of high-resolution modeled atmospheric fields. *J*. *Atm*. *Ocean*. *Tech*. **33**, 303–311, doi:10.1175/JTECH-D-15-0132.1 (2015).

[43] Pauluis O, Garner S (2006). Sensitivity of radiative-convective equilibrium simulations to horizontal resolution. J. Atm. Sci..

[44] Murphy, D. M. Rare temperature histories and cirrus ice number density in a parcel and a one-dimensional model. *Atm*. *Chem*. *Phys*. **14**, 13013–13022, http://www.atmos-chem-phys.net/14/13013/2014/, doi:10.5194/acp-14-13013-2014 (2014).

[45] Dinh T, Podglajen A, Hertzog A, Legras B, Plougonven R (2016). Effect of gravity wave temperature fluctuations on homogeneous ice nucleation in the tropical tropopause layer. Atm. Chem. Phys..

[46] Bacmeister J, Eckermann S, Tsias A, Carslaw K, Peter T (1999). Mesoscale temperature fluctuations induced by a spectrum of gravity waves: A comparison of parameterizations and their impact on stratospheric microphysics. J. Atmos. Sci..

[47] Herzog A, Vial F (2001). A study of the dynamics of the equatorial lower stratosphere by use of ultra-long-duration balloons. J. Geophys. Res..

[48] Gayet J (2004). Cirrus cloud microphysical and optical properties at southern and northern midlatitudes during the inca experiment. J. Geophys. Res..

[49] Barahona, D. *et al*. Development of two-moment cloud microphysics for liquid and ice within the nasa goddard earth observing system model (geos-5). *Geosc*. *Model Dev*. **7**, 1733–1766, http://www.geosci-model-dev.net/7/1733/2014/, doi:10.5194/gmd-7-1733-2014 (2014).

[50] Phillips VT (2013). Improvements to an empirical parameterization of heterogeneous ice nucleation and its comparison with observations. J. Atm. Sci..

[51] Rienecker, M. *et al*. *The GEOS*-*5 Data Assimilation System* - *Documentation of Versions 5*.*0*.*1*, *5*.*1*.*0*, *and 5*.*2*.*0*., vol. 27 of *Technical Report Series on Global Modeling and Data Assimilation* (NASA Goddard Space Flight Center, Greenbelt, MD, USA, 2008).

[52] Joos, H., Spichtinger, P., Gayet, J. & Minikin, A. Orographic cirrus in the global climate model echam5. *J*. *Geophys*. *Res*. **113** (2008).

[53] Sato K (1990). Vertical wind disturbances in the troposphere and lower stratosphere observed by the mu radar. J. Atmos. Sci..

[54] Wang M, Penner J (2010). Cirrus clouds in a global climate model with a statistical cirrus cloud scheme. Atmos. Chem. Phys..

[55] Gary BL (2008). Mesoscale temperature fluctuations in the southern hemisphere stratosphere. Atm. Chem. Phys..

[56] Muhlbauer A, Kalesse H, Kollias P (2014). Vertical velocities and turbulence in midlatitude anvil cirrus: A comparison between *in situ* aircraft measurements and ground-based doppler cloud radar retrievals. Geophys. Res. Lett..

[57] Muhlbauer, A. *et al*. Impact of large-scale dynamics on the microphysical properties of midlatitude cirrus. *J*. *Geophys*. *Res*.: *Atmospheres* **119**, 3976–3996, http://dx.doi.org/10.1002/2013JD020035, doi:10.1002/2013JD020035 (2014).

[58] DeMott P (2003). Measurements of the concentration and composition of nuclei for cirrus formation. Proc. Natl. Acad. Sci. USA.

[59] Field P (2006). Some ice nucleation characteristics of asian and saharan desert dust. Atmos. Chem. Phys..

[60] Kärcher B, Lohmann U (2002). A parameterization of cirrus cloud formation: homogeneous freezing of supercooled aerosols. J. Geophys. Res..

[61] Barahona D, Nenes A (2008). Parameterization of cirrus formation in large scale models: Homogeneous nucleation. J. Geophys. Res..

[62] Hoyle C, Luo B, Peter T (2005). The origin of high ice crystal number densities in cirrus clouds. J. Atmos. Sci..

