# Supplementary material for: Direct estimation of the global distribution of vertical velocity within cirrus clouds
Source: Sci Rep. 2017 Jul 28;7:6840. doi: 10.1038/s41598-017-07038-6 (PMC5533806; doi:10.1038/s41598-017-07038-6)
Supplement: Supplementary file 1 — Supplementary Material [file 41598_2017_7038_MOESM1_ESM.pdf]

Direct estimation of the global distribution of vertical velocity within cirrus clouds- supplementary material

Donifan Barahona[1,*], Andrea Molod[1] and Heike Kalesse[2]

[1]Global Modeling and Assimilation Office, NASA Goddard Space Flight Center, Greenbelt, MD, USA
[2]Leibniz Institute for Tropospheric Research, Leipzig, Germany
[*]donifan.o.barahona@nasa.gov

ABSTRACT

This document provides supplementary figures accompanying the main text.

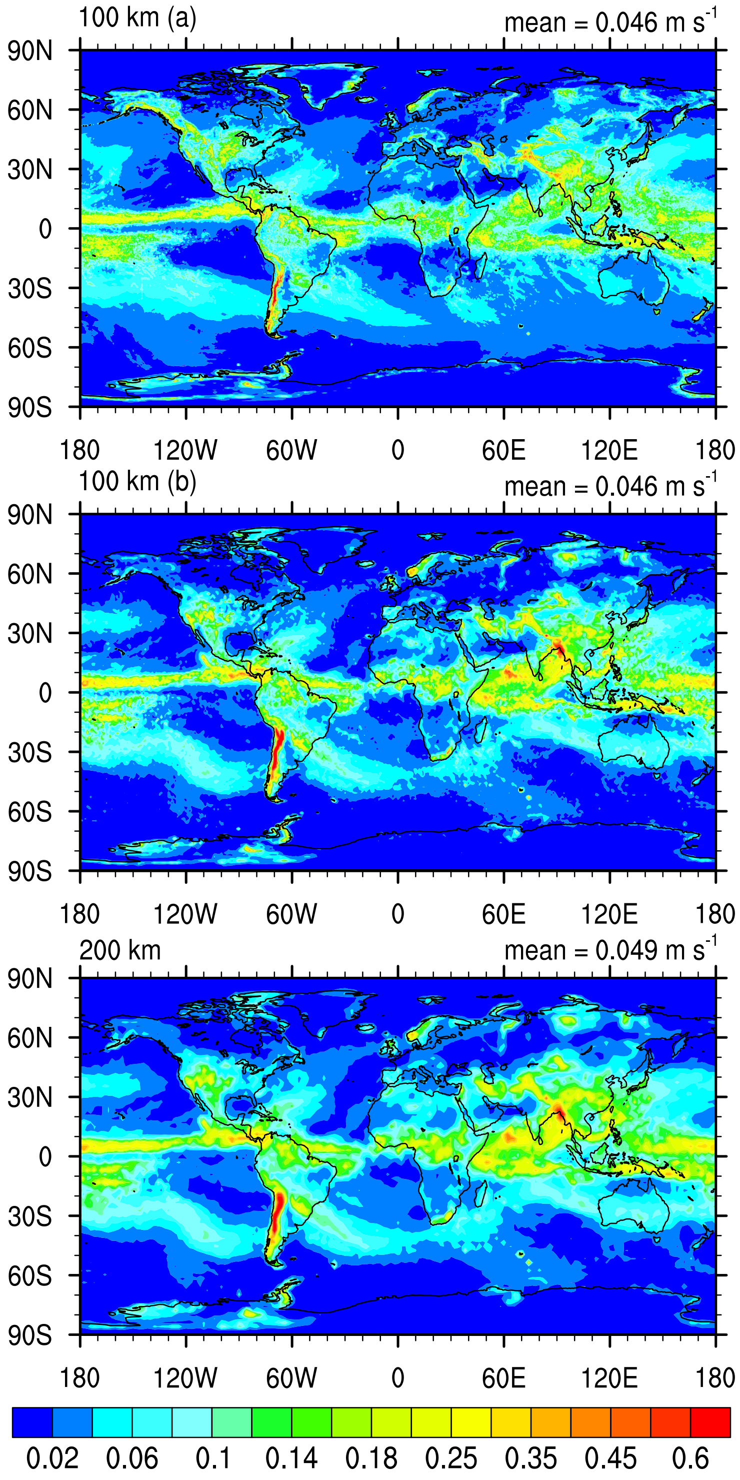

Figure S1. Monthly mean standard deviation in vertical velocity (m s^{-1}) at pressure level 250 hPa for May 2006 at horizontal resolutions of 100 km and 200 km. The top panel was calculated using the 7 km global output. The middle and bottom panels were calculated using 3.5 km global output. Maps generated using the NCAR Command Language (Version 6.3.0) Software. (2016). Boulder, Colorado: UCAR/NCAR/CISL/TDD. http://dx.doi.org/10.5065/D6WD3XH5.

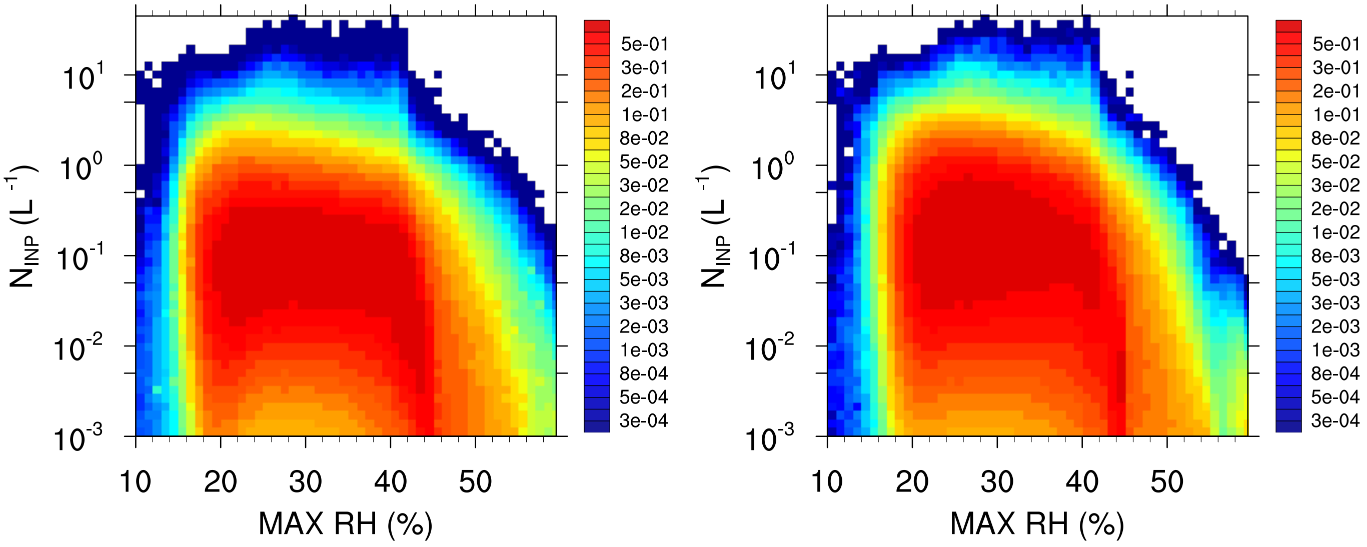

Figure S2. Frequency distribution of the concentration ice nucleating particles, N_{INP}, as a function of the maximum in-cloud relative humidity with respect to ice. GEOS-5 output was used over a 2-year subset (2005–2006) and 100 km horizontal resolution. Left panel: Global. Right panel: Northern Hemisphere.

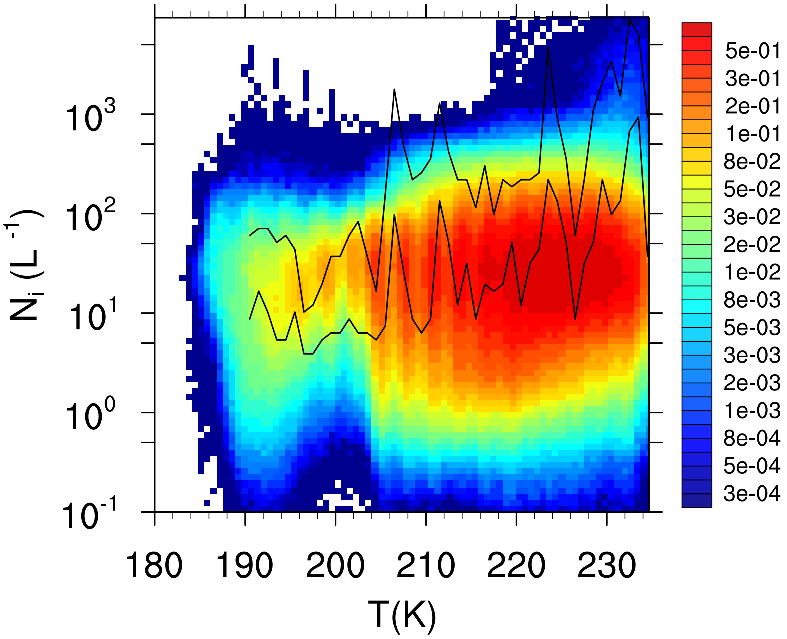

Figure S3. Frequency distribution of in-cloud ice crystal number concentration as a function of temperature for the Northern Hemisphere, from GEOS-5 output over a 2-year subset (2005–2006). Solid lines represent the 25 % and 75 % quantiles from a compilation of field campaign observations[1].

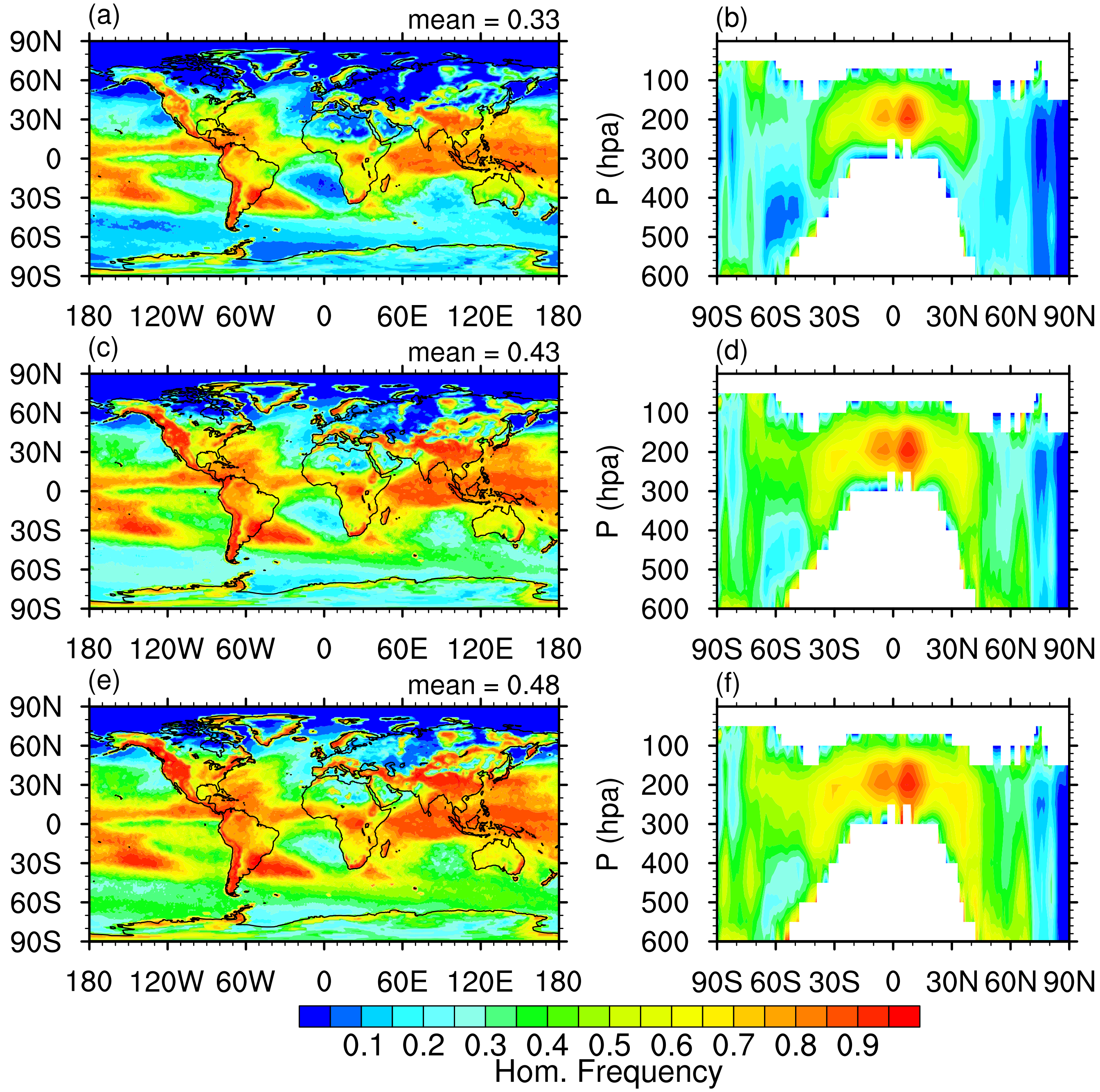

Figure S4. Global distribution of the frequency of cirrus events with HOM nucleation producing 90% (a, b), 50% (c, d), and 20% (e, f) of ice crystals. Left panels: vertically weighted by cloud fraction. Right panels: zonal mean. Maps generated using the NCAR Command Language (Version 6.3.0) Software. (2016). Boulder, Colorado: UCAR/NCAR/CISL/TDD. http://dx.doi.org/10.5065/D6WD3XH5.

References

1. Krämer, M. *et al.* Ice supersaturation and cirrus cloud crystal numbers. *Atmos. Chem. Phys.* **9**, 3505–3522 (2009).
